# Acknowledgement to Reviewers of *Children* in 2014

**DOI:** 10.3390/children2010037

**Published:** 2015-01-12

**Authors:** Children Editorial Office

**Affiliations:** MDPI AG, Klybeckstrasse 64, CH-4057 Basel, Switzerland

The editors of *Children* would like to express their sincere gratitude to the following reviewers for assessing manuscripts in 2014: 

Alexander, StephanieHoward, JustinePfeifer, Luzia IaraAnderson, James A.Hummell, Donna S.Plantz, DianeBaranowski, TomHutchon, DavidPole, JasonBernstein, AaronKatholi, Benjamin R.Poplawski, NicolaBhatia, SmitaKean, JamesPost-White, JaniceBrenner, MichelleKenney, LisaPritchard, SheilaBreuner, CoraKim, S. SamuelRajaraman, PreethaBrown, MelanieKjellgren, CeciliaRamsay, J. RussellCalderón-Garcidueñas, LilianKlorer, GussieRennie, SteveCampos, Maria CeliaKniepeiss, DanielaRobien, KimChandra, JoyaKovats, SariRossie, Jean-PierreChemaitilly, WassimKretzmann, MarkRussell, WendyChen, WeiKrysinska, KarolinaRusy, Lynn M.Choi, LillianKunin-Batson, AliciaSahler, OllieChoonara, ImtiLachenmeier, Dirk W.Schamberger, MagdalenaCrews, JonathanLambert, VeronicaSchmölzer, Georg M.Crittendon, JulieLee, Evon BSee, Ching MeyDevine, KatieLester, StuartSeidel, BastianEdwards, StevenLoren, Alison W.Sencer, SusanEsaki, NinaLowen, DeborahShen, YipingEtzel, Ruth A.Marsenic, OliveraSlaughter, James C.Euser, SaskiaMayfield, Margie I.Sluss, DorothyEvans, SubhadraMcDonald, MariaStagnitti, KarenFothergill, AliceMertens, AnnStillwell, TerriFrazer, PaigeMetitieri, TizianaStores, GregoryFriedl, ErikaMeyer, JillStott, RobinFusco, CarolineMitchell, DianeTanes, ZeynepGassin, ElizabethMontgomery, HughTassone, Rosalie F.Gils, Jan VanMorris, JaneTemple, Dr. Jennifer L.Glanzman, Marianne MMosby, TerezieTrenkle, Ph.D., BernhardGodfrey, LesliMulder, Renée LUrback, StaceyGoldberger, DannyNewmark, SanfordValentine, JaneGolden, William LNicklas, Theresa A.von Baeyer, Ph.D., CarlGray, PeterNoonan, CurtisWagner, CarolGreen, Daniel M.Oates, JohnWaterston, TonyGulden, ToreOdland, Jon ØyvindWatson, MichaelHanson, CorrineOrtibus, Els L.Weydert, JoyHébert, Michèle L. J.Pacey, AllanWoodyer, TaraHendershot, EleanorPaul, BriannaYeh, Ann MingHenderson, TaraPellis, SergioYeh, Jennifer M.Hewes, Jane



